# Osteomyelitis of the Jaw in COVID-19 Patients: A Rare Condition With a High Risk for Severe Complications

**DOI:** 10.3389/fsurg.2022.867088

**Published:** 2022-07-01

**Authors:** Ana Kvolik Pavić, Vedran Zubčić

**Affiliations:** ^1^ Department of Maxillofacial and Oral Surgery, Osijek, Croatia; ^2^ Faculty of Medicine, Osijek Josip Juraj Strossmayer University of Osijek, Osijek, Croatia

**Keywords:** osteomyelitis, jaw, surgical complication, COVID-19, concomitant infection, healthy, odontogenic

## Abstract

Osteomyelitis of the jaw is an uncommon infection that arises from the flora of the oral cavity or sinuses and affects immunocompromised and polymorbid patients. Treatment includes surgical debridement and long regiments of broad-spectrum antibiotics. We present three cases of complicated jaw osteomyelitis presented with concurrent COVID-19 infection, including only two reported cases of odontogenic COVID-related osteomyelitis. The two mandibular cases were patients in their 30s with no comorbidities. The first case was an asymptomatic COVID-19-positive patient who developed an odontogenic infection after tooth extraction that was complicated by the second bout of abscess formation and localized osteomyelitis. The second case was a COVID-19-positive patient with an odontogenic infection that presented as airway compromise due to trismus and neck edema, which required an emergency tracheotomy. He developed osteomyelitis of the mandibular ramus that was reconstructed with a titanium plate. The third case was a polymorbid post-COVID-19 patient who developed a protracted infection of the maxillary sinus that resulted in the loss of an eye, destruction of the maxilla, palate, and parts of nasal cavum, and oronasal incontinence. The defect was reconstructed with a microvascular anterolateral thigh flap. We hypothesize that COVID-19-related immune dysfunction and microvascular changes contributed to osteomyelitis in our patients.

## Introduction

Osteomyelitis is an infection of the bone that arises from the bone marrow and rarely affects healthy individuals (1). It can be caused by hematogenous (mostly in the pediatric population) or contiguous spread of the infection (after trauma, joint replacement surgery, or odontogenic infection) or be associated with vascular deficiency (such as in patients with poorly regulated diabetes) (2). The jawbones are predisposed to infections because of a primary contaminated environment of the oral cavity, periodontal pockets that harbor multitudes of anaerobic bacteria, a thin mucosal lining that adheres to the periosteum, and the existence of teeth and pneumatization of the maxilla. An insult, such as infection or trauma, causes an increase in intramedullary pressure, which leads to reduced blood flow and immune response, further facilitating the spread of the infection (2). Mandibular osteomyelitis is usually odontogenic but can also be caused by trauma, certain medications (such as bisphosphonates), and radiation therapy of the neck. Maxillary osteomyelitis can also result from maxillary sinusitis (1, 3).

Severe acute respiratory syndrome coronavirus 2 (SARS CoV-2) virus quickly swept through the world and became a pandemic only months after its first isolation and identification (4). It is a respiratory virus that can cause every clinical scenario ranging from a patient who is an asymptomatic carrier to severe respiratory distress that could be fatal in an immunocompromised patient (4, 5). Hyperinflammation caused by SARS CoV-2 infection can influence and aggravate pre-existing conditions (6), but it is questionable whether an asymptomatic disease impacts the healthy (7). With millions of people becoming infected with this novel virus, the aftermath of infection on both healthy and compromised patients remains to be elucidated.

In this paper, we present three cases of osteomyelitis of the jaw complicated with SARS CoV-2 infection, including only two reported cases of odontogenic COVID-related osteomyelitis of the jaw. Cases are listed from least (1) to most severe (3). Possible implications of concomitant SARS CoV-2 on infection of the bone are discussed.

## Cases

### Case 1

A 28-year-old otherwise healthy female presented to the Emergency Maxillofacial Service for swelling in the left submandibular region that began a week after the extraction of tooth 38. She tested negative for SARS CoV-2 virus upon admittance and was treated with extraoral incision and drainage in general anesthesia and a course of intravenous antibiotics (Clindamycin and Gentamycin). An intraoperative swab showed that the infection caused by *Prevotella denticola*, a commensal oral bacterium. The recovery was uneventful, and the patient was discharged 4 days postoperatively.

**Figure 1:**
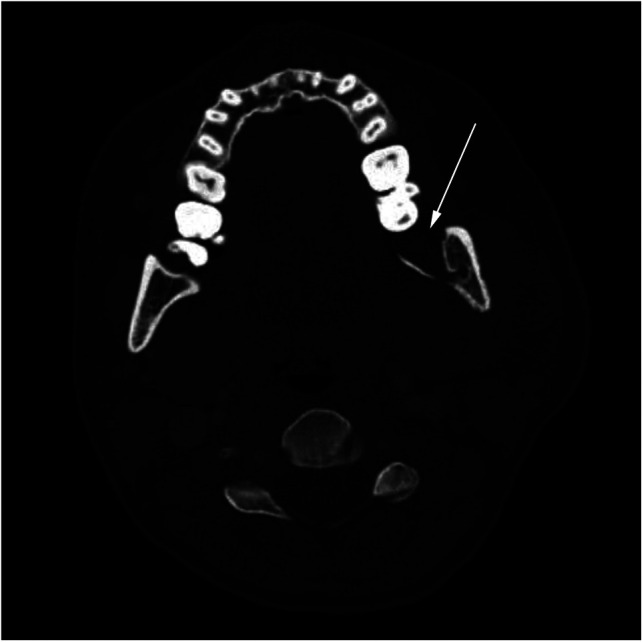
Preoperative axial CT scan of the neck at the C2 level showing osteomyelitis in region 38 of the mandibular ramus. The affected side shows loss of cortical margin (arrow) and soft tissue swelling.

Three weeks later, the patient returned to Emergency Maxillofacial Service with swelling in the operated region. Again, she tested negative for the SARS CoV-2 virus. CT scan showed lamellar perimandibular purulent collection, cellulitis of soft tissue, and osteomyelitis surrounding the alveolus of the extracted tooth (Figure 1). She received another extraoral incision, drainage, and the second batch of intravenous antibiotics. On the second postoperative day, she tested positive for COVID-19 and was transferred to the Surgical COVID Unit, where her recovery was uneventful. She was discharged on the fifth postoperative day and had no SARS CoV-2-related symptoms whatsoever.

The follow-up included panorex scans and scintigraphy scans that showed remodellation of the angular region of the mandible and regression of the inflammation.

### Case 2

The patient was a 29-year-old male patient with no previous ailments. He had a 10-day history of diffuse toothache, perimandibular, and parotid swelling before presenting to the Emergency Maxillofacial Service. His airway was compromised by trismus and edema of soft tissues of the neck. He tested positive for SARS CoV-2 virus upon hospital admittance.

CT showed massive perimandibular, parapharyngeal, parotid, temporal, and infratemporal abscess caused by an inflammation of tooth 48. He was treated by emergency tracheotomy, extraoral incision, and drainage, followed by a course of parenteral antibiotics (Penicillin B, Metronidazole, and Gentamycin). The patient needed additional oxygenation and spent one day in the COVID Respiratory Unit. He was discharged on the 10th postoperative day to house quarantine. Microbiological swab samples of the purulent collection taken during surgery were sterile.

**Figure 2:**
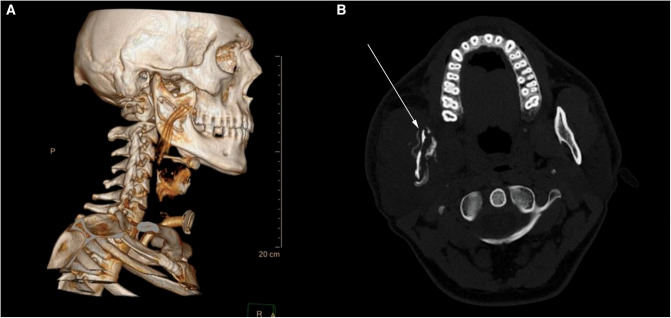
Axial CT scan of the neck at the C1 level after the relapse of the infection. (**A**) 3D reconstruction showing sequestrum of bone with a corrugated drain properly placed above the osteomyelitic site. (**B**) Cross section of the osteomyelitic mandibular ramus—bone is thin with loss of both medulla and cortex. A reconstructive plate was placed so the mandible could withstand masticatory forces.

A week later, the patient returned to the Emergency Maxillofacial Service with another bout of swelling in the parotido-masseteric region. The CT scans confirmed relapse of abscess in previously infected regions. He was treated with a reincision, drainage, and intravenous antibiotics (Clindamycin and Gentamycin). Postoperative scintigraphy showed inflammation of the ramus of the mandible, and CT confirmed sequestrum formation in the ramus (Figure 2). We performed sequestrectomy and debridement of the affected bone, followed by reconstruction and osteosynthesis with a reconstructive titanium plate.

Follow-up scintigraphy scans showed regression of the inflammatory processes of the mandible, and the patient was referred to the dental service for further treatment.

### Case 3

The patient is 71-year-old male with a previous history of multiple myeloma, diabetes mellitus, arterial hypertension, and chronic renal insufficiency who was recovering from SARS CoV-2 bilateral pneumonia. He was discharged from the COVID unit 2 weeks before presenting to the Emergency Maxillofacial Service with redness and swelling around the left eye. He had a loss of sight in the affected eye, left-sided facioparesis, crusting in the left nasal cavum, and maceration of the left side of the hard palate. His lab results upon admittance are as follows: white blood cell count (WBC) 33.9×10^9^/L, CRP 216.7 mg/L, and elevated liver and kidney function tests. The patient's glucose was elevated at 18.1 mmol/L and HbA1c was 9.9%, although the patient reported having better control of his glucose levels before this infection. His initial CT scans showed an abscess in the left maxillary sinus and ethmoids. He was treated with incision and drainage of the orbital and paranasal abscess—both incisions yielded copious amounts of pus—and put on a course of intravenous Ceftriaxone and Clindamycin. Intraoperative swabs showed *Enterococcus faecium*, *Enterococcus fecalis* VRE, and *Candida lusitanie* infection. After the fungal infection was confirmed, Fluconazole was added to his medications.

**Figure 3:**
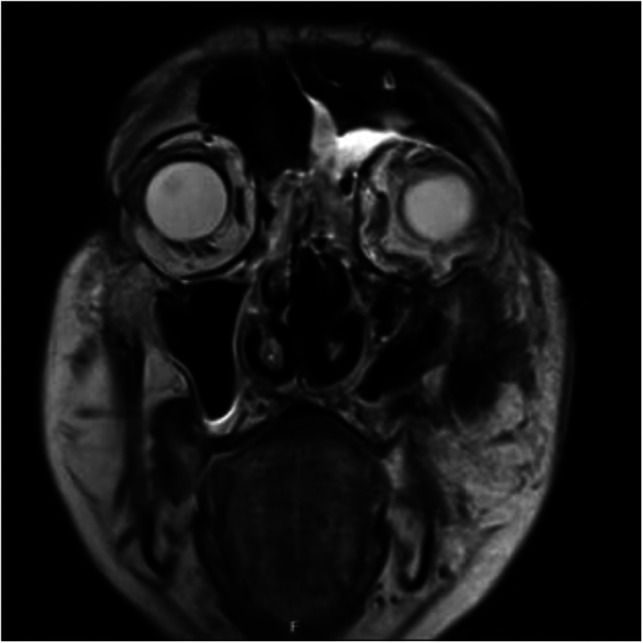
Coronal MRI of the orbits and sinuses taken on the fourth day after hospital admittance: cellulitis of the left orbit with hyperintense signal in preseptal and postseptal areas. Inflammatory thickening of the mucosal lining of the left frontal sinus, ethmoid cells, and maxillary sinus.

After the initial incision and drainage, the patient's lab results remained elevated (WBC 30.5 × 10^9^/L, CRP 167.9 mg/L), and his overall status deteriorated. Repeated CT and MRI scans (Figure 3) showed progression of the inflammation that was treated surgically with debridement of necrotic tissue, irrigation, and iodoform gauze packing. A total of six operations in general anesthesia amounted to exenteration of the orbit, partial ethmoidectomy, debridement of the hard and soft palate, the vomer, and sequestrectomy of the maxillae. Histopathological analysis of the excised tissue showed inflammation, necrosis, and copious amounts of hyphae formation.

He was discharged after 29 days of hospital stay with an acrylic obturator plate that served to close the defect left in the hard palate. Part of the necrotic maxilla remained visible through the cutaneous fistula.

**Figure 4:**
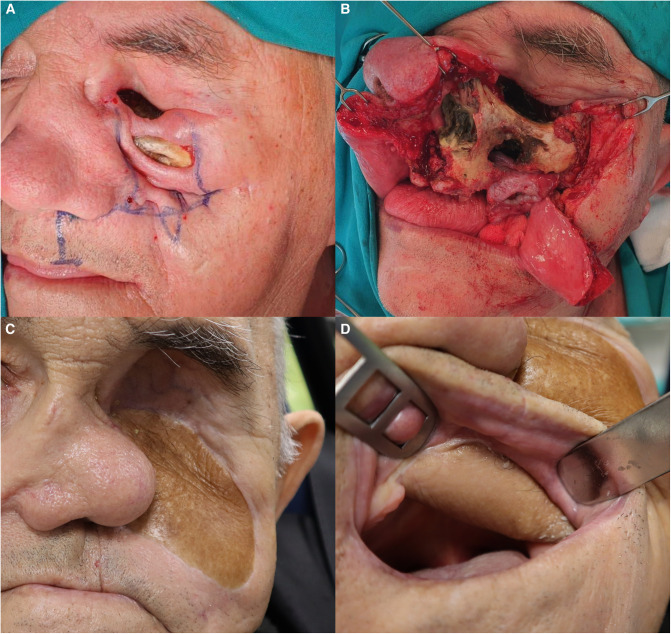
Reconstruction of the defect. (**A**) Preoperative planning with modified Weber–Ferguson incision. (**B**) Exposed necrotic bone. (**C**) 9-month follow-up. The orbital and facial defect is closed, and maxillary dead space is obliterated with the bulk of the ALT flap. The nasal pyramid is leaning toward the operated side because the bone and cartilage support has been removed. (**D**) ALT flap lining the palatal defect.

He was readmitted after 2.5 months for reconstruction. His main complaints were oronasal incontinence when drinking or eating soft food and foul odor. Due to multiple comorbidities, we choose not to reconstruct the bony aspect of the maxilla. Instead, he received a microvascular anterolateral tight flap that served to line the orbital, nasal, and palatal defect and separate the nasal cavum from the mouth (Figure 4). The goal was to achieve a better quality of life and oronasal continence. He was discharged on the ninth postoperative day, and his recovery was uncomplicated by infection or dehiscence.

At a 6-month follow-up, he had no complaints in the donor or receiving region.

## Discussion

Osteomyelitis of the jaw is becoming a relatively rare entity due to better oral hygiene, dental care, and the widespread use of antibiotics. Nevertheless, during the COVID-19 pandemic, there has been an increasing number of reports of osteomyelitis of facial bones (8) as well as an increased number of deep neck infections (9).

The particularity of jaw bones is their relationship with teeth and pneumatized spaces of maxillary sinuses. Both teeth and sinuses can harbor a subclinical infection that becomes acute in the case of a drop in the immune status of the host or emergence of a more virulent strain of pathogen (3, 10). As such, jawbones are at constant risk for contiguous spread of the infection from surrounding foci.

Osteomyelitis begins with inflammation of the cancellous bone. After the initial bacterial nidus establishes itself in the bone, leucocytes begin excreting inflammatory factors that cause increased blood flow in the acute phase of osteomyelitis (10). The bone cannot expand, so minuscule blood vessels in the bone become congested. This contributes to relative hypoperfusion in the center of infection and reactive hyperemia in the outskirts. Hypoxic areas become inaccessible to the body's immune system as well as antibiotics, so segments of bone become necrotic and form sequestrums (2).

Local hypoperfusion causes lower bioavailability of antibiotics in inflamed bone, while the penetrance of antibiotics inside the purulent collection is limited (3). Pus formed around the necrotic bone cannot be cleared without incision, drainage, and debridement. A study on animal models showed that surgical drains and irrigation of the osteomyelitic site improve bone healing compared with just debridement (11). Even in cases where no pus can be obtained upon incision, some surgeons argue that early incision and drainage diminishes tissue pressure and improves circulation, thus speeding the recovery (12, 13).

It is widely reported that SARS CoV-2 causes an immune system dysfunction that could swing the course of the disease from asymptomatic or mild to severe (7, 14). Clinical studies show that SARS CoV-2 infection can cause a depletion of certain strains of leucocytes, which reduce the hosts’ ability to fight off an infection, although there are no studies proving the direct link (15). A retrospective study on ICU patients in Wuhan showed a decrease in CD4 and CD8 lymphocytes, which are responsible for coordinating the immune response in the fight against viral and bacterial infection, as well as cancer, in much the same way as HIV infection does (16). This mechanism could be responsible for atypical clinical features of osteomyelitis in our patients.

Besides damaging the immune system, COVID-19 causes vasculopathy that could contribute to the clinical features of our patients. Procoagulative status in COVID-19 is mediated by endothelial lesions with fibrinogen and complement deposits (17–19). These sites could promote occlusive thrombi, which lead to tissue ischemia and inadequate perfusion (19), which is already stunted in areas of chronic jaw bone infection (20). Decreased blood flow caused by COVID-19 vasculopathy with increased vascular permeability and perivascular tissue edema could be an aggravating factor for the development of osteomyelitis.

“Happy hypoxemia” is one of the presentations of COVID-19 disease where a patient is profoundly hypoxemic but does not show signs of respiratory distress (14, 21). Areas of hypoperfusion and systemic hypoxia favor anaerobic bacteria, which are usually abundant in oral microbiota (22, 23). Also, the mandible is physiologically more susceptible to the infection because its blood supply is lower compared to other facial bones (3).

Fauci et al. have reported an estimation that 80% of infection remains undocumented (5), either because the patient is asymptomatic and does not know he is infected or the symptoms are so mild that they do not require medical assistance. It is not surprising that there is currently no study on the immune response of asymptomatic or mild COVID-19 patients. Some subclinical immune system dysfunctions may predispose asymptomatic COVID-19 patients to bacterial superinfections or concomitant infection.

The COVID-19 pandemic has resulted in unforeseen medical consequences that cannot all be traced back to the COVID-19 disease. As patients are advised to avoid nonessential medical visits, it is sometimes difficult to determine when a medical issue is becoming “essential.” A study on orthodontic patients in treatment found a 9-week delay in appointments during the pandemic, with one-third of patients experiencing orthodontic emergencies (24). Our department saw a decrease in outpatient visits to only one-third compared with prepandemic time (25). Similar effects are published across medical specialties (26–29). As medical practitioners, we must strive to provide the same level of care to our patients as we did before the pandemic.

## Conclusion

In this case series, we presented three patients with an unusual course of head and neck infection that resulted in osteomyelitis of the jaw. All patients tested positive for the SARS CoV-2 virus shortly before or during the infection. In these cases, immune dysfunction and tissue hypoxia caused by COVID-19 disease could be aggravating factors for concomitant infection. Early surgical intervention (incision, drainage, debridement) and targeted antibiotic treatment are crucial in resolving acute osteomyelitis.

We hope this case series will raise questions about the impact of mild COVID-19 on concomitant infections and the immunological system of infected patients that will be answered in future studies.

## Data Availability

The original contributions presented in the study are included in the article, further inquiries can be directed to the corresponding author.
